# Self-Inflicted Total Amputation of the External Genitalia as a Psychological Repercussion of the COVID-19 Pandemic: A Case Report

**DOI:** 10.7759/cureus.32725

**Published:** 2022-12-20

**Authors:** Amal AL-Fahdi, Tamadhir Al-Mahrouqi, Ibrahim AlZain, Harith AL-Aamri, Salim AL-Huseini

**Affiliations:** 1 Department of Behavioral Medicine, Sultan Qaboos University, Muscat, OMN; 2 Department of Psychiatry, Oman Medical Specialty Board, Muscat, OMN; 3 Department of Psychiatry, Al Masarra Hospital, Ministry of Health, Muscat, OMN

**Keywords:** case report, oman, quarantine, self-amputation, covid-19

## Abstract

During the coronavirus disease 2019 (COVID-19) pandemic, mandatory quarantine has interrupted everyday social life, leaving many individuals feeling confined and lonely, with increased rates of suicide and suicidal behavior. Genital self-mutilation (GSM) is a rare phenomenon that typically occurs in the context of severe mental illness. The following case report describes the first case of total self-inflicted genital amputation in a healthy man who had no prior history of mental or medical conditions. This case poses challenges for psychiatric diagnosis and treatment. We report a case of a 52-year-old male with no significant medical and psychiatric history who was admitted to a tertiary care hospital after attempting suicide by self-inflecting a total amputation of his external genitalia due to fear of COVID-19 infection. The diathesis-stress linked with the tribulations of the current pandemic was highlighted as well as other possible contributory factors and psychopathology behind his clinical presentation.

## Introduction

The global coronavirus 2019 (COVID-19) outbreak at the end of 2019 has triggered widespread worries, fear, and stress, all of which are reasonable and natural reactions to the world’s unusual circumstances [1]. Many people’s worries have been heightened by regular World Health Organization (WHO) press briefings regarding the disease's spread and the various methods to combat it such as wearing masks and hand hygiene. However, quarantine, physical distancing, living alone, and the uncertainties associated with the COVID-19 disease are all known risk factors for psychological distress and suicide [2,3]. According to a survey in Norway, people who adhered to government-initiated social distancing measures during the COVID-19 pandemic were primarily afflicted by loneliness [4]. Individuals who experience loneliness are more likely to develop psychological conditions such as depression, anxiety, suicidal ideation, and parasuicidal behavior [5,6]. There are many different methods by which individuals commit suicide, some of which are more lethal than others. The most frequent methods include using a weapon, jumping from a great height, hanging, drowning, overdosing, poisoning, and cutting [7].On the other hand, genital self-mutilation (GSM) is one of the least common methods of suicide, having only been documented on a handful of occasions in the medical literature [8]. Self-mutilation of the external genitals is common in individuals with a psychiatric history, particularly psychosis, and it occurs in all racial groups, cultures, and religions [9,10]. In Kingsor syndrome, delusions and hallucinations are connected with religious themes [9,11]. Other psychiatric disorders, such as personality disorders, substance use disorder, and gender dysphoria, have been linked to genital self-mutilation, but it is less common in anxiety and mood disorders [12]. To contribute to the growing body of literature on the effects of the COVID-19 pandemic on self-harm and suicidal behavior, we report the first case of a man, who was previously healthy and had never had a history of physical or mental illness, completely amputating his external genitalia on his own in an attempt to commit suicide owing to fear of COVID-19 infection.

## Case presentation

A 52-year-old male, married with three children, had a good relationship with his wife with normal sexual behavior. He was previously physically and mentally healthy and presented to the emergency department (ED) at a tertiary hospital in Muscat, Oman, after self-amputation of the whole of his external genitalia. Ten days before the presentation, he had been in contact with several COVID-19-positive individuals in his residence. Following this discovery, he became increasingly concerned and distressed and contemplated the possibility of contracting COVID-19 infection. His local healthcare authorities tracked him down and advised him to remain in self-isolation. He was quarantined for 10 days and, due to boredom and a lack of social interaction, experienced low mood and reported feeling lonely throughout that time. Moreover, he reported feeling hopeless and helpless and that he was preoccupied with the idea that he would die soon as a result of COVID-19 infection, therefore he chose to commit suicide by cutting his external genital. He denied having a specific reason for cutting his genitalia rather than another area of his body. He claimed he only did it to kill himself. He had total amputation of his external genital (the shaft of his penis, scrotum, and testes). He took all the necessary precautions to prevent the failure of his suicidal attempt, therefore, he chose to execute his plan early in the morning so that no one could rescue him. He was found unconscious on the toilet floor by the man who delivered the lunch meal several hours after the incident. He was rushed to the emergency room and scheduled for surgery. They discovered that the penis and both testes had been entirely sliced at the root, with the cord severed at the external ring. The scrotum was likewise removed entirely. Both the spermatic cord and corporal blood vessels were bleeding. The wound was closed and his surgical recovery was uneventful. The psychiatric evaluation did not reveal previous self-harming behaviors, suicidal attempts, substance use, body image disturbance, or sexual identity disturbances. There were no psychotic or schizophrenia symptoms in the past or present. The mental status examination revealed an anxious mood, he was preoccupied with COVID-19 infection, with suicidal ideation, he was alert and oriented to time and place and his insight and judgment were intact. His hemoglobin was 10.5 g/dl while thyroid function and complete metabolic profile were all normal. The urine drug screen resulted negative, and an X-ray of the pelvis revealed no abnormalities. He scored 12/27 on the Patient Health Questionnaire-9 (PHQ-9) scale. At the time of admission, there was no history of cough, shortness of breath, fever, or other respiratory symptoms. After finding that he had tested negative for COVID-19, he felt relieved. And during his hospitalization, the frequent psychiatric evaluations revealed depression, and the diagnosis according to the Diagnostic and Statistical Manual of Mental Disorders, Fifth Edition (DSM-5), was major depressive disorder, single episode, moderate. We conducted patient-centered care and encouraged the patient's active collaborative and shared decision-making in his management. And the patient opted to begin only with supportive psychotherapy, deep breathing exercise, close-one-to-one observation, and no pharmacological intervention were initiated. Moreover, social services were alerted, and social study and social support were provided. After six weeks of admission, the patient was fit for discharge and he had no suicidal thoughts or plans. He followed outpatient psychiatric services for four weeks, and then he was referred based on his request to a local psychiatrist in his neighborhood to continue psychotherapy.

## Discussion

In previous case reports, the majority of individuals with genital self-mutilation have been linked to psychotic episodes, particularly religious delusion this was not the case in our patient [13]. In fact, the distinguishing characteristics in our case were a sense of isolation, hopelessness, and helplessness, as well as the presence of suicidal intent in the absence of any psychotic experiences and influence by cultural and religious, beliefs.

Some people believe that the genital has a rich blood supply and that cutting it will result in immediate death. Self-inflicted genital injury is extremely rare, to say the least, outside of severe mental diseases such as psychosis or temporary states caused by drug or alcohol intoxication; it is practically non-existent. That said, there was a time in history when self-castration, or gelding, as it was called, was seen as a sign of extreme devotion or self-purification in the service of one’s lord. *For there are some eunuchs, which were so born from their mother's womb: and there are some eunuchs, which were made eunuchs of men: and there be eunuchs, which have made themselves eunuchs for the kingdom of heaven's sake. He that can receive it, let him receive it *[14]. 

Alanna Skuse 2018 examined examples of such behavior; she reports that self-castrators, driven by emotional stressors, sought to attack the body, which they may have felt was divorced from the mind and self and thus could not be governed by it. The acts were taken to keep the 'self' from succumbing to the body's desire [15]. Another argument is that some men could resort to this extreme act again to gain control of a situation. Our patient denied any conflict, religious or otherwise, that lead him to this act. At the least, not through conscious contemplation as he maintained. There remains the distressing notion of coming face to face with one’s imminent mortality [16]. During his time in isolation, the gentleman explained that he did believe that his life would end soon and that the thought of dying pushed him toward suicide. This is not unheard of; indeed, cancer patients are known to be at a higher risk of suicide, for instance, compared to the general population [17].

Death wishes and the desire to 'hasten death' through euthanasia or medically assisted suicide are marked by depression, anxiety, and the feeling of being hopeless and alone [18]. Symptoms of helplessness and hopelessness were displayed by our patient. We remain with the modus and why our patient chose castration as a means to an end in his pursuit of a swift death. When interviewed, he did not have a recollection or explanation for this, nor unfortunately was he available for further comment. We are left therefore to speculate as to the reason for this choice. Throughout antiquity and the ancient world leading up to today, the phallus has been seen as the symbol of life and the sign of manhood in many if not all cultures [19]. It is not that far-fetched a notion, therefore, that the patient, coming face to face with severe illness or even death, had a moment of existential despair. With a complete loss of hope of ever coming out on the other side of his dilemma, he proceeded to sever his manhood. Our patient is unable to see a future of a meaningful life in any shape or capacity (father, husband, or man). All roles are vital to his being and cannot be seen separate from his existence.

## Conclusions

This case highlights the psychological impact of COVID-19 infection among healthy individuals. Compounded with the added stress of the prospect of one’s mortality, this can lead, as we saw in our patient, to extreme behavior. Therefore, clinicians must consider the wide variety of psychiatric conditions and psychosocial factors that underlie GSM.
